# Understanding uptake of the COVID-19 vaccination among the homeless: A mixed methods evaluation

**DOI:** 10.1371/journal.pone.0312617

**Published:** 2025-01-08

**Authors:** Grace Phillips, Emmy Racine, Anna Marie Naughton, Julieann Lane, Patricia M. Kearney

**Affiliations:** 1 School of Public Health, University College Cork, Cork, Ireland; 2 Adult Homeless Integrated Team, Health Service Executive, Ireland; University of Foggia: Universita degli Studi di Foggia, ITALY

## Abstract

**Background:**

Ensuring effective access to vaccinations for people experiencing homelessness is crucial to protecting the health of a vulnerable, yet often overlooked population. Reaching this goal takes more than a one size fits all approach. This study evaluates how a dedicated health team collaborated with multiple agencies to register and deliver the COVID-19 vaccine to people experiencing homelessness.

**Methods:**

This is a mixed methods study co-designed with the Adult Homeless Integrated Team, a multi-disciplinary team who work with local agencies to provide care to people experiencing homelessness in Cork, Ireland’s second largest city. Quantitative data collected at the point of vaccine registration described socio-demographics of the population. To explain the quantitative findings, eleven agencies involved in provision of homeless services were invited to participate in interviews. A manager in each of the agencies acted as a gatekeeper to clients. Interviews explored experiences with the pandemic and the delivery (staff) or receipt (clients) of the COVID-19 vaccine. Interviews were recorded and transcribed, transcriptions were thematically analysed.

**Results:**

There were 728 vaccine doses administered to people experiencing homelessness during the first roll-out of vaccines; 401 first doses and 325 second doses. Of those who received a vaccine, the majority were male (76%), and more than half were Irish (55%). Ten semi-structured interviews, seven staff members and three clients, were conducted. There were three themes that provided further insights into the quantitative findings: *Adapting to unprecedented times*, *Misinformation causing vaccine hesitancy* and *The importance of building relationships*.

**Conclusions:**

This study provides valuable insights into how a multidisciplinary approach resulted in a successful well received vaccination programme among a traditionally hard to reach group.

## Background

Homelessness affects more than 100 million people globally leaving many people without a place to live that is safe, supports their needs and is in decent condition [1]. When the Coronavirus disease 2019 (COVID-19) pandemic struck in March 2020 there were 9,907 people homeless in Ireland [2]. At the start of the pandemic, additional accommodation was sourced for people accessing emergency accommodation reducing the number of individuals sleeping rough and reducing the risk of transmission of COVID-19. However, from May 2021 homeless numbers rose steadily, so that by July 2022 there were more than 10,568 people experiencing homelessness nationally [2].

Ensuring effective access to vaccinations for people experiencing homelessness is crucial to protecting the health of a vulnerable, yet often overlooked population [3]. Routine vaccine uptake for illnesses such as tuberculosis, hepatitis and seasonal influenza has previously been low in this population [4]. Similarly, despite evidence demonstrating that COVID-19 can be significantly damaging for those experiencing homelessness, vaccination rates are lower than the general population [5, 6]. Reasons for this include, factors imposed by homelessness such as scheduling appointments and arranging transportation to vaccination clinics [7]. Personal factors such as mistrust of healthcare providers, fear of needles, and concerns that vaccines will cause illness may discourage people experiencing homelessness from accessing vaccinations [8].

People experiencing homelessness are deemed a hard-to-reach group, due to their life circumstances and uncertain living arrangements [9]. While the Centre for Disease Control recommended Johnson and Johnson (single dose) vaccine for people experiencing homelessness to optimise uptake [10], in Ireland, the type of vaccine administered varied in different regions of the country. The purpose of this research is to evaluate the impact of the multidisciplinary approach taken by the health team in establishing a bespoke clinic and vaccinating the homeless population of Cork City.

## Methods

### Study design

This explanatory sequential mixed method study includes a description of the vaccination roll-out, a descriptive quantitative data analysis of sociodemographic characteristics of people experiencing homelessness and qualitative data collection and analysis with service providers and clients. The explanatory sequential mixed methods design involves first collecting and analysing quantitative data, then using qualitative data to explain the quantitative results, in this study quantitative data collected at the point of COVID-19 vaccine registration was explained by semi-structured interviews with both staff and clients involved in the COVID-19 vaccine rollout. The study was co-designed with the Adult Homeless Integrated Team (AHIT) in Cork City. AHIT is a sub-group of the Health Service Executive (HSE) Social Inclusion service. The HSE is the national health service in Ireland. The HSE Social Inclusion service aims to reduce inequalities in health and improve access to mainstream and targeted health services for vulnerable and excluded groups in Ireland [11].

### Co-design

AHIT approached the School of Public Health to carry out a process evaluation of the vaccine rollout to people experiencing homelessness in Cork City. Initially, phone calls took place between the lead researcher and coordinator of the AHIT. After a site visit to a vaccination clinic in phase one of the COVID-19 vaccine rollout on the 13^th^ of May, a meeting was organised between the research team (lead researcher [GP] and senior academic [PK] and the AHIT (Clinical lead [AMN] and Homeless Services coordinator [JL]), via MS Teams. This meeting was led by the AHIT, who provided an in-depth description of the vaccination process from planning to delivery of the first vaccine. Initially, a process evaluation involving quantitative data was envisaged. Following discussion of the evaluation goals, the importance of gaining a deeper understanding of the perspectives of staff and clients was identified. It was therefore decided to include qualitative data collection and analysis. Over five meetings and one site visit between May 2021 and January 2022 the research design was further developed. These meetings also involved developing invitation letters, consent forms, topic guides for interviews, and deciding on and aiding the recruitment process.

### The COVID-19 vaccine rollout process

A detailed description of the process of the vaccination rollout for the homeless population was obtained from a lead administrator in the Adult Homeless Integrated Team. Administrators from HSE Social Inclusion service contacted statutory and non-statutory homeless services in Cork City to determine the number of vaccines required for the rollout. Social care staff were given a deadline to return numbers by, and a template was distributed to collect key data on those interested in receiving a vaccine. Pending this information, the team, comprised of clinical and non-clinical staff, completed training on becoming peer vaccinators and on COVAX system use. All interested clients were registered on COVAX prior to attending vaccination clinics.

Once dates, times and clinic locations were decided, clinics were organised in two of Cork’s busiest homeless hostels to enable greater coverage and accessibility. It was important that all clinics had adequate space for vaccinators to administer the vaccine, ensuring confidentiality and respect throughout the process. Hostel managers identified members of staff to welcome clients, accompany clients from vaccinator to waiting area, manage the flow of people throughout the clinic and clean communal areas.

A timetable template was created in consultation with both homeless hostels involved, based on the clinics, vaccinators, and clients. A multidisciplinary approach with input from social care staff, shelter managers, key workers and managers from statutory and non-statutory homeless services was applied in the allocation of times. Social care staff in this step were aware of their clients’ routines, which informed decisions about timing of appointments.

Agencies were asked to engage and support persons to attend clinics for their appointment and aid in the completion of COVAX registration forms e.g., addressing language barriers or literacy issues, prior to their appointment. Completed forms were cross checked at clinics with the information previously uploaded to the COVAX system. Administrators colour coded forms to let vaccinators know that all information had been checked and clients were eligible for vaccination. Staff regularly counted vaccine numbers and clients to avoid wasting of vaccines. Staff created a *standby list* and drew from this when appointments were not filled. This process was replicated for second dose vaccines. First dose clinics took place on the 10th, 11th and 13th of May and second dose clinics took place on the 8th, 9th and 10th of June. Clients who wished to receive a first dose vaccine after the first rollout of clinics, were welcome to do so in the June rollout. An additional clinic was arranged on the 23rd of June. Any remaining clients who were not vaccinated in this rollout and wished to be vaccinated were referred to City Hall, the main site for delivery of vaccines to the general population.

### Quantitative data analysis

Any adult in Cork City registered as homeless in May 2021 was eligible for inclusion in the analysis. Data were extracted from the HSE Social Inclusion COVID-19 vaccine register of people experiencing homelessness. These data were collected for use in COVAX, a global initiative aimed at ensuring equitable access to COVID-19 vaccinations. Variables included age, gender, nationality, language, vaccine status and accommodation status. The data were anonymised and provided to the study team in an encrypted password protected excel file. Descriptive statistics were used to summarise the data. The statistical software Stata Version 14 (StataCorp, TX, USA) was used in this analysis.

### Qualitative data collection and analysis

#### Recruitment of Health and social care staff

Participants from eleven agencies including the HSE Social Inclusion service and statutory and non-statutory homeless services in Cork were interviewed using a semi-structured approach. A contact list of managers involved in the vaccine rollout was compiled by the HSE Social Inclusion service and provided to the study team. An email invitation was sent to the managers of each agency inviting them to participate in qualitative interviews and a reminder email was sent after two weeks. A snowball sampling approach was applied in recruitment of staff, participants who agreed to participate then extended the invitation to their colleagues. Recruitment began on the 21^st^ of February 2022 and ended on the 30^th^ of March 2022.

#### Recruitment of people experiencing homelessness

Any adult registered as homeless in May 2021 was eligible to participate. Participants were recruited from eleven statutory and non-statutory homeless services in Cork City. An invitation was sent by email to all managers of these agencies. Managers acted as gatekeepers, extending the invitation to their clients. It was emphasized to participants that participation in the study was entirely voluntary and would have no impact on the service they receive from HSE Social Inclusion service or the agency in which they reside. Recruitment began on the 22^nd^ of March and ended on the 30^th^ of April 2022.

### Data collection and analysis

Interviews were conducted with health and social care staff via MS Teams and recorded using MS Teams recording feature. Face to face interviews were conducted with clients of statutory and non-statutory homeless services in Cork City in the location where clients were residing. The interviews were recorded using a Dictaphone.

A topic guide, which was structured around research objectives and quantitative results, mainly questions on reasons for high COVID-19 vaccine uptake, guided the interviews. There were separate topic guides for staff and clients which explored perspectives on the barriers and facilitators of the COVID-19 vaccine rollout to people experiencing homelessness.

Interview recordings were transcribed, anonymised, and thematically analysed. The thematic analysis applied the Braun and Clarke approach: familiarization with data, generation of codes, searching for themes, reviewing themes, and defining and naming themes [1, 12]. Initial code development took place using (NVivo 11 software) and then manually until saturation was reached. Data saturation was determined when no new codes were developed. These initial codes were refined into categories and sorted into potential themes and subthemes. The relevant coded data extracts were collated into themes.

### Ethical considerations

This study received ethical approval from the Social Research Ethics Committee and the School of Public Health Ethics Committee. Full written consent was obtained from all interview participants.

## Results

In May 2021, there were 414 people accessing emergency accommodation in Cork City, this figure does not include those accessing addiction treatment centres or domestic violence centres [13]. From May-June 2021, the AHIT in partnership with statutory and non-statutory homeless services administered 726 Pfizer vaccine doses to people experiencing homelessness in Cork City: 401 first doses and 325 second doses resulting in a dose two uptake of 81%. Overall, 34 people did not return for their second dose vaccine; 21 were uncontactable, seven received their second dose elsewhere, one person emigrated, four people were in prison, and one person was in hospital.

Of those who received a COVID-19 vaccine, three quarters were male (n = 305; 76%) and more than half were Irish (n = 222; 55%). The majority were residing in homeless hostels and B&Bs at the time of vaccination. The sociodemographic characteristics of those who received at least one COVID-19 vaccine are summarised in Table 1.

**Table 1:** Population demographics.

Variable	N	%	
**Gender**			
Male	305	76	
Female	97	24	
**Nationality**			
Irish	222	55	
English	3	0.75	
Polish	11	3	
Other	15	4.5	
Unknown	150	37	
**Language**			
English	200	55	
Polish	7	2	
Other	8	2	
Unknown	166	41	
**Accommodation Status**			
Homeless Hostels & B&Bs in Cork City	255		
Youth Homeless Residential Services	11		
Family Homeless Services	34		
Treatment Residential Centre	42		
Other Homeless Services	60		
**Year of Birth**	**Min**	**Max**	**Mean**
	2003	1951	1983

### Qualitative data

Interviews took place between February and May 2022. In total, seven staff members (five female and two male) and three clients (three male) agreed to take part in semi-structured interviews. Six worked in homeless services, one worked in social inclusion services, and one worked in an administrative roll within the Health Service Executive. Of clients who took part, all were residing in homeless services in Cork City. Interviews took between 20–60 minutes.

Seven themes were developed from the qualitative data relating to participants’ experiences and attitudes towards the COVID-19 vaccine rollout (Table 2).

**Table 2:** Themes, sub-themes and quotes.

Themes	Sub-themes	Quotes
Adapting to Unprecedented Times	• “Military Precision”–Social Care Staff were Best Placed to Rollout This Intervention • “The Pfizer was the kind of gold standard”–The Influence of the Pfizer Vaccine • “I have to give credit to them”–Client’s Praise for Staff Organisation Efforts	• *Our facilities manager here*, *had a tough time initially accessing masks [staff]*. • *We tested once a week and we isolated clients in one of our residential units*, *we were fortunate to form good links with the Mercy Hospital and the labs in the Mercy Hospital [staff]*. • *People were getting their appointments*, *getting their vaccines and they were getting their certs*, *everything was military precision [staff]*. • *It’s a transient population*. *People*, *going to tenancies and maybe the tenancy breaks down and they get a letter sent to the next address of an appointment in hospital and they miss the appointment [staff]*. • *Some people would have been presenting under the influence of alcohol or something else*. *And uh*, *I suppose we know how to deal with people in that situation [staff]*. • *It was really stressful for us when we were trying to run a project anyway with all the other things that go on*, *on a day-to-day basis and then being landed very last minute with*, *you need to get 40 to 50 people over to location by tomorrow kind of thing [staff]*. • *For some of our clients like it’s*, *you know*, *having to travel somewhere to do something will just be a put off straight away*. *Like so if things kind of come to us*, *it would be better [staff]*. • *If I meet somebody and encourage them to get a vaccine*, *I need to be able to stand over that and say*, *I know because I’ve got the Pfizer and I was alright*, *my arm didn’t fall off [staff]*. • *It wasn’t about Johnson and Johnson or Pfizer or Astra Zeneca [staff]*. • *[The Nurse] registered me straight away*, *within talking to her I would have had my first vaccine within a week and then she would have followed up with the next one [client]*. • *They were fantastic in [the vaccination centre] I have to give credit to them; it was all organised*, *you went in you saw the nurse*, *you were treated with the upmost respect*, *you sat down for your 20 minutes and then you go*. *It was totally dignified [client]*.
The importance of building relationships	• “Nobody to support them”—Overcoming Social Isolation • “Relationships are key”- The Importance of a Trusting Staff-Client Relationship	• *Some people have very low social networks*. *They have nobody to support them*, *nobody to remind them nobody to encourage them [staff]*. • *Carefully monitoring*, *we’re they going to go [to their vaccination appointment]*? *Did you go*? *If they did go*, *how did they get on*? *How they were afterwards; you know advising them to rest and all that kind of support that came with that [staff]*. • *There are family issues*, *perhaps their focus is trying to recover from addiction or there are lots and lots of difficulties [staff]*. • *It was very much the role of the key worker then to speak to them in relation to that vaccine and the importance of getting the vaccine and so that relationship was actually key [staff]*. • *The staff and nurses were perfect*, *the doctor is the best person to give advice [client]*. • *It was the nurse in [homeless hostel]*, *I would have known her a good few years so she was in touch about getting it done and it was done very fast*, *very efficient [client]*. • *The key workers saying lads ye have to get this done*, *and fair play to them for doing that [client]*.
Overcoming Misinformation	• ‘They’re putting trackers in you’- Misinformation causing Vaccine Hesitancy	• *They were reading a lot of stuff on social media and things like that they were advising them not to take it up [staff]*. • *We had to spend a lot of time chatting to them about that [misinformation] and saying*, *where did you hear that*? *Where’d you get that information*? *How do you know that’s true*? *Trying to get them to challenge themselves and where they got the information from and how do they know it’s true*? *[staff]* • *I was saying I’m not getting that thing done at all*, *they’re putting trackers in you [client]*. • *I was a bit worried about it at the start*, *as everyone else was [client]*.

### Themes

#### Adapting to unprecedented times

At the outset of the pandemic, staff encountered significant challenges with the pandemic which motivated their response to the COVID-19 vaccine rollout. Three staff members reported difficulty obtaining personal protective equipment, while others reported the need for increased testing and isolation facilities within their organisation. Staff working in residential organisations described the implementation of many safeguards, including a reduction in capacity, to ensure the safety of staff and clients.

#### *Military precision*–Social care staff were best placed to rollout this intervention

Both staff and clients described the *military precision* with which the COVID-19 vaccine rollout was organised and communication being central to its success. Social care staff individually contacted their clients to offer them the COVID-19 vaccine. During these interactions, they provided all the necessary information about the vaccines and answered any questions that arose.

Six staff members praised the clinic’s organisation and flexibility, which was vital given the transient nature of people experiencing homelessness. This bespoke clinic allowed people to get their vaccination at a later date, or time if they were unable to attend an appointment, staff highlighted the importance of this, as it is not always easy for people experiencing homelessness to reschedule appointments. Additionally, some staff reported to have accommodated clients who may have been under the influence of substances, which may not have been the case in an external organisation. Similarly, one client emphasized how the flexibility of a bespoke clinic benefited them.

While feedback from the COVID-19 vaccine rollout was generally positive, some staff reported areas for improvement. One participant reported increased stress while registering clients for the COVID-19 vaccine and the time given to notify their clients of the vaccine rollout was too short. One staff member suggested running additional clinics to ensure that everyone who wanted to receive the vaccine had the opportunity to do so.

#### *The Pfizer was the kind of gold standard*–The influence of the Pfizer vaccine

Considering the higher stated efficacy of the Pfizer vaccine (two dose) and based on the existing trusting relationship with clients, a decision was made to offer the same (Pfizer) vaccine to people experiencing homelessness as was being delivered nationally to the general population. Four staff members emphasized the importance of using the Pfizer vaccine, as this allowed them to refer to their own experience when providing information to their clients, with one participant reporting their clients urge to wait for the Pfizer vaccine. However, two staff members had the view that the type of vaccine offered had no effect on COVID-19 vaccine uptake. All three clients reported the type of vaccine used would not have influenced their decision to take the COVID-19 vaccine.

#### *I have to give credit to them*–Client’s praise for staff organisation efforts

Three clients expressed their satisfaction with the COVID-19 vaccine rollout, citing the high level of organisation and flexibility demonstrated by the staff. Clients reported that staff maintained constant communication, arranging appointments, sending reminders, and ensuring that clients were comfortable throughout the process. Overall, all three clients had a positive experience with the vaccination process. While one client appreciated the convenience of having the clinic located nearby.

### The importance of building relationships

#### *Nobody to support them*—Overcoming social isolation

All staff members interviewed recognised the challenges faced by people experiencing homelessness and stressed the importance of providing accurate information regarding the COVID-19 vaccine and supporting their decisions. Staff reported showing compassion towards those who had already received the vaccine and provided necessary care and assistance.

Staff highlighted the difficulties faced by people experiencing homelessness such as, mental health disorders, substance abuse, high stress lives, social isolation, and overall poor health. Staff stressed how this can generally cause people experiencing homelessness to miss appointments, and without help, they may not be able to reschedule appointments or prioritise an issue that requires attention.

#### *Relationships are key—*The importance of a trusting staff-client relationship

Health and social care staff, particularly key workers, described their strong and trusting relationship with their clients, and reported this as fundamental in the high vaccine uptake. The importance of the trusting relationship between staff and clients was also emphasized in client interviews, with all three clients citing it as a key factor in their decision to get vaccinated. One client even mentioned that they would not have known about the vaccine if it had not been brought to the hostel in which they were residing.

### Overcoming misinformation

#### *They’re putting trackers in you—*Misinformation causing vaccine hesitancy

Five staff members encountered vaccine hesitancy while providing information on the COVID-19 vaccine. They recalled instances where clients were reading misinformation on social media, which contradicted the evidence provided by staff members. Staff members stressed the importance of educating clients about COVID-19 vaccines while also listening to their concerns about the vaccine. All three clients were initially concerned about the COVID-19 vaccine with one client reporting to have believed conspiracy theories that they had heard from others.

## Discussion

### Summary of main findings

This mixed method evaluation of the COVID-19 vaccine rollout to people experiencing homelessness demonstrates how an innovative and flexible approach to vaccine delivery can achieve very high uptake in a traditionally hard to reach population. Over 400 people experiencing homelessness including 305 males (77%) and 97 females (24%), majorly residing in hostels and B&Bs received the COVID-19 vaccine. Interviews with both staff and clients further explained the reasons for high uptake in this population. The multidisciplinary and collaborative approach with social care staff having an administrative and educational role in recruiting and educating clients maximised COVID-19 vaccine access and uptake. Interviews with staff and clients identified the importance of organisation and flexibility in delivery of the COVID-19 vaccination programme, and the importance of staff-client relationships in overcoming misinformation, social isolation and other difficulties faced by people experiencing homelessness.

### Comparisons to existing literature

Achieving high vaccination coverage among vulnerable groups is critical to preventing morbidity and mortality from COVID-19. This study is one of few that add to the body of evidence surrounding COVID-19 vaccination programmes for people experiencing homelessness. A survey conducted on “The Maximising Uptake Programme”, a COVID-19 vaccination programme which was rolled out across Southwest England to increase vaccine uptake in vulnerable groups, showed that 39% of respondents preferred to attend the local clinic rather than an NHS facility and 67% did not want to attend a GP practice or mass vaccination centre to get vaccinated [9]. This study, similar to our work, demonstrates the importance of reaching out through a targeted bespoke clinic.

In some parts of the United Kingdom, mass vaccination programmes took place in homeless shelters, in other areas, charities and frontline workers took it upon themselves to organise vaccination hubs [7, 14]. Canada also recognised the importance of prioritizing people experiencing homelessness as pop-up vaccination clinics were rolled out in homeless shelters across the country [7]. Similar to Cork’s vaccine rollout, they took on a multi-disciplinary approach, involving shelter staff, community outreach workers and local healthcare organisations [7]. However, there is no data on the overall uptake in these populations.

Our findings are consistent with previous studies [9, 15] which suggest that effective communication and education from trusted individual can increase COVID-19 vaccine uptake in this group. Similarly, knowing a trusted individual who has received a vaccine also had a positive impact on vaccine uptake [16, 17]. The involvement of social care staff and key workers with existing relationships with clients has been an essential part of vaccinating people experiencing homelessness [7].

### Strengths

This study’s strengths include the co-design with the AHIT who played a key role in managing the COVID-19 vaccine rollout, providing key insights into the process of the vaccination rollout. The experience and expertise of the AHIT in working with people experiencing homelessness informed many decisions from use of language, to where interviews would be held.

Another strength of the study was the use of semi-structured interviews with staff and clients ensured that key areas were covered while also allowing for the flexibility to explore relevant themes that arose through dialogue. The use of thematic analysis provided an opportunity to delve into the data and develop themes without preconceived notions.

The use of the explanatory sequential mixed methods design provided a more comprehensive and nuanced view of vaccine uptake in people experiencing homelessness. The quantitative data offered a sociodemographic view of the population, while the qualitative data provided rich insights and context to explain why COVID-19 vaccine delivery was so successful.

Finally, our findings highlight the benefits of using existing health and homeless services that are already accessed and trusted by people experiencing homelessness to deliver health services to a traditionally hard to reach population.

### Limitations

There are limitations to this study. Firstly, the small size and restricted geography in Cork City limits generalizability to people experiencing homelessness in other locations or in other health care systems. Participants were recruited through statutory and non-statutory homeless services; potentially excluding people experiencing homelessness who have not engaged with these organisations.

Interviews took place up to one year after the vaccination programme was rolled out. Due to the transient nature of people experiencing homelessness, many had moved on from the service in which they were residing. Those who chose not to be vaccinated were not included in the study, thus we were unable to identify barriers to COVID-19 vaccination. Multiple emails were sent to statutory and non-statutory homeless services inviting participants for interview, however there was a relatively low uptake. Most staff interviewed provided positive feedback on the vaccine rollout, staff who declined to participate may have had different views.

All quantitative data included in the study was collected by the HSE, this data therefore is not validated, and a large proportion of data is missing. Additionally, the HSE cyber-attack occurred in the middle of this vaccine rollout, therefore the HSE had limited access to resources and previous information they had collected on clients, forcing them to move to hard copies, losing data. This meant that we were unable to compare clients who received the vaccine with those who did not, or to explore what characteristics changed over time between Dose one and Dose two. Therefore, the authors were only able to analyse data which provided a sociodemographic description of those who received a COVID-19 vaccine. Additionally, given the vulnerability of people experiencing homelessness there is often a reluctance to providing personal information therefore it was not required that they answer questions on nationality and language. This resulted in a large proportion of missing data.

### Ethical considerations

Due to the nature of the study, clients may have found answering questions about the COVID-19 vaccine difficult. Therefore, all interviews were conducted on the premises in which participants were residing with staff members of the agency in proximity if any issues arose. All participants were advised of their right to discontinue participation at any point during the study. Participants were assured that their data would be confidential, and any report of the study will not include identifiable information.

### Implications

This study provides insights into COVID-19 vaccination uptake and attitudes among those experiencing homelessness in Cork City. To ensure that health interventions reach the *hard to reach*, it is necessary to include health, social inclusion, and homeless services in the decision-making process because they have built trusting relationships with clients and are trained to deal with and understand the needs of people experiencing homelessness. It is critical for staff to provide clients with clear health messaging and to support their decisions. Our findings demonstrate the need for a client centred and multidisciplinary approach to delivering health interventions to vulnerable population’s thereby empowering people experiencing homelessness to make health-related decisions.

### Conclusion

This mixed method evaluation of the COVID-19 vaccine rollout to people experiencing homelessness provides valuable insights into how a multidisciplinary approach resulted in a successful well received vaccination programme among a traditionally hard to reach group. Our study emphasises the importance of organisation and flexibility in scheduling appointments for people experiencing homelessness. To promote vaccination in this population, a strong support system and a trusting staff-client relationship is essential.

## Supporting information

S1 File Recruitment information. (DOCX)

## References

[1] Herre Bastian and Arriagada Pablo (2023)—“Homelessness” Published online at OurWorldinData.org. Available from: ’https://ourworldindata.org/homelessness’

[2] Department of Housing, Planning & Local Government. Homelessness Report March 2020. Available from: https://www.gov.ie/pdf/?file=https://assets.gov.ie/73702/40be8abcf4db4f1cb7dc6269c6d32928.pdf#page=null

[3] Centers for Disease Control and Prevention. Community, Work, and School. 2020. Available from: https://www.cdc.gov/coronavirus/2019-ncov/community/homeless-shelters/vaccine-faqs.html

[4] Iacoella C, Ralli M, Maggiolini A, Arcangeli A, Ercoli L. Acceptance of COVID-19 vaccine among persons experiencing homelessness in the City of Rome, Italy. Eur Rev Med Pharmacol Sci. 2021 Apr;25(7):3132–5. doi: 10.26355/eurrev_202104_25568 33877682

[5] Tsai J, Wilson M. COVID-19: a potential public health problem for homeless populations. Lancet Public Health. 2020 Apr;5(4):e186–7. doi: 10.1016/S2468-2667(20)30053-0 32171054 PMC7104053

[6] Rogers JH, Cox SN, Hughes JP, Link AC, Chow EJ, Fosse I, et al. Trends in COVID-19 vaccination intent and factors associated with deliberation and reluctance among adult homeless shelter residents and staff, 1 November 2020 to 28 February 2021 –King County, Washington. Vaccine. 2022 Jan 3;40(1):122–32. doi: 10.1016/j.vaccine.2021.11.026 34863618 PMC8590934

[7] Paudyal V, Racine M, Hwang SW. COVID-19 vaccination amongst persons experiencing homelessness: practices and learnings from UK, Canada and the US. Public Health. 2021 Oct 1;199:e2–3. doi: 10.1016/j.puhe.2021.08.015 34548161 PMC8407945

[8] Metcalfe SE, Sexton EH. An Academic-Community Partnership to Address the Flu Vaccination Rates of the Homeless. Public Health Nurs. 2014;31(2):175–82. doi: 10.1111/phn.12088 24741686

[9] Flook M, Grohmann S, Stagg HR. Hard to reach: COVID-19 responses and the complexities of homelessness. Lancet Respir Med. 2020 Dec 1;8(12):1160–1. doi: 10.1016/S2213-2600(20)30446-X 32979307 PMC7511203

[10] Pagaduan J. National Alliance to End Homelessness. 2022 [cited 2022 Aug 25]. What are the Latest Insights on Vaccination for People Experiencing Homelessness? Available from: https://endhomelessness.org/blog/what-are-the-latest-insights-on-vaccination-for-people-experiencing-homelessness/

[11] Health Service Executive. About Social Inclusion. Available from: https://www.hse.ie/eng/about/who/primarycare/socialinclusion/about-social-inclusion/about-social-inclusion.html

[12] Braun V, Clarke V. Using thematic analysis in psychology. Qual Res Psychol. 2006 Jan;3(2):77–101.

[13] Department of Housing, Planning & Local Government. Homeless Report May 2021. Available from: https://www.gov.ie/en/publication/fbd27-homeless-report-may-2021/

[14] Evans R. Covid: “Let charities vaccinate the homeless.”. 2021. Available from: https://www.bbc.com/news/uk-wales-55984840

[15] Balma B, Vasilakos L, Osman I, Elgonda A, Gewirtz O’Brien JR. COVID-19 vaccine attitudes among youth experiencing homelessness: a qualitative analysis with opportunities for action. BMC Public Health. 2023 Aug 31;23(1):1672. doi: 10.1186/s12889-023-16413-0 37648987 PMC10469469

[16] Sapienza A, Falcone R. The Role of Trust in COVID-19 Vaccine Acceptance: Considerations from a Systematic Review. Int J Environ Res Public Health. 2022 Dec 30;20(1):665. doi: 10.3390/ijerph20010665 36612982 PMC9819668

[17] Longchamps C, Ducarroz S, Crouzet L, Vignier N, Pourtau L, Allaire C, et al. COVID-19 vaccine hesitancy among persons living in homeless shelters in France. Vaccine. 2021 Jun 8;39(25):3315–3318. doi: 10.1016/j.vaccine.2021.05.012 Epub 2021 May 12. ; PMCID: PMC8114835. 34011464 PMC8114835

