# Supplementary material for: Understanding uptake of the COVID-19 vaccination among the homeless: A mixed methods evaluation
Source: PLoS One. 2025 Jan 8;20(1):e0312617. doi: 10.1371/journal.pone.0312617 (PMC11709251; doi:10.1371/journal.pone.0312617)
Supplement: S1 File — (DOCX) [file pone.0312617.s001.docx]

# **Supplementary Material – Study Information**

**Appendix A**

**Information Sheet for Staff**

**INFORMATION SHEET**

**
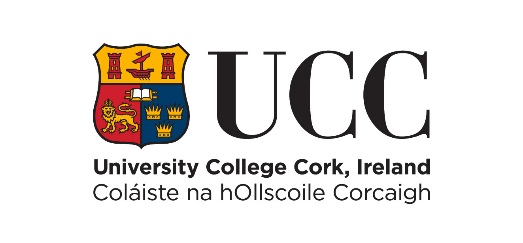
**

**Title of the Study.** Process Evaluation of the COVID19 Vaccination Programme for the Homeless Population of Cork City.

**Purpose of the Study.** The purpose of the study is to investigate the COVID19 vaccine rollout to the homeless population of Cork City, which will help to inform future interventions for vulnerable populations and achieve a long-term goal of making health services more accessible to difficult to reach groups. This research is being conducted as part of a research project at the School of Public Health, UCC.

**What will the study involve?** The study will involve collecting anonymized data from those registered homeless in Cork. This will be completed in two steps, firstly data on age, gender, location, and uptake will be obtained prior to vaccination. Secondly, face to face interviews will take place with service users and staff. Participants will be asked about their experience with the vaccine rollout, this data will then be anonymized.

**Why have you been asked to take part?** You have been asked because you played a role in the rollout of the COVID19 vaccine to the homeless population of Cork City.

**Do you have to take part?** Participation in this study is voluntary, you will not be included in this study in the absence of a signed consent form. You will have the option of withdrawing before the study commences or discontinuing after data collection has started. All data will be anonymized.

**Will your participation in the study be kept confidential?** Yes. I will ensure that no clues to your identity appear in the study or any other publications or outputs from this research. Any extracts from what you say that are quoted will be entirely anonymous.

**What will happen to the information which you give?** The data will be kept confidential for the duration of the study, available only to me and my research supervisor. It will be securely stored on UCC secure server (NAS drive). On completion of the project, they will be retained for minimum of a further ten years and then destroyed.

**What will happen to the results?** The results will be seen by the research team. We will also use findings in an anonymized way in publications and presentations to share the information with healthcare professionals, scientists and policymakers and the wider community.

**What are the possible disadvantages of taking part?** I don’t envisage any negative consequences for you in taking part.

**What if there is a problem?** At the end of the procedure, I will discuss with you how you found the experience and how you are feeling. If you subsequently feel distressed, I have included a list of support services below:

**YourMentalHealth**

The YourMentalHealth information line is a phone service you can call any time 24/7.

A member of their team can tell you about:

- - the mental health supports and services available to you
  - how to access different services provided by the HSE and our funded partners
  - opening hours

Freephone: 1800 111 888

The YourMentalHealth information line is not a counselling service.

**Samaritans**

They provide a listening service to anyone who needs it, no matter what you are going through.

Freephone: 116 123

**Pieta House**

They provide support for people who are suicidal and people who self-harm.

Freephone: 1800 247 247

Face-to-face support

For face-to-face support contact your GP.

You can also search this list of mental health supports and services near you.

**Urgent help**

Call 999 or 112 and ask for the ambulance service if you or someone you know needs emergency help.

**Who has reviewed this study?** This study has been reviewed by the Social Research Ethics Committee of UCC.

**Any further queries?** If you need any further information, you can contact me: Grace Phillips ([grace.phillips@ucc.ie](mailto:grace.phillips@ucc.ie)) contact the HSE contact person, Julieann Lane on (066) 7184535.

If you agree to take part in the study, please sign the consent form overleaf.

**Appendix B**

**Information Sheet for Service Users**

**INFORMATION SHEET**

**
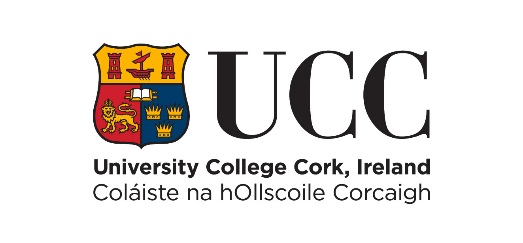
**

**Title of the Study.** Process Evaluation of the COVID19 Vaccination Programme for the Homeless Population of Cork City.

**Purpose of the Study.** The purpose of the study is to investigate the COVID19 vaccine rollout to the homeless population of Cork City, which will help to inform future interventions for vulnerable populations and achieve a long-term goal of making health services more accessible to difficult to reach groups. This research is being conducted as part of a research project at the School of Public Health, UCC.

**What will the study involve?** The study will involve collecting anonymized data from those registered homeless in Cork. This will be completed in two steps, firstly data on age, gender, location, and uptake will be obtained prior to vaccination. Secondly, face to face interviews will take place with service users and staff. Participants will be asked about their experience with the vaccine rollout, this data will then be anonymized.

**Why have you been asked to take part?** You have been asked to participate in this study because you completed the HSE COVID19 vaccination programme.

**Do you have to take part?** Participation in this study is voluntary, you will not be included in this study in the absence of a signed consent form. You will have the option of withdrawing before the study commences or discontinuing after data collection has started. All data, including interviews, will be anonymized.

**Will your participation in the study be kept confidential?** Yes. I will ensure that no clues to your identity appear in the study. Any extracts from what you say that are quoted will be entirely anonymous.

**What will happen to the information which you give?** The data will be kept confidential for the duration of the study, available only to me and my research supervisor. It will be securely stored on UCC secure server (NAS drive). On completion of the project, they will be retained for minimum of a further ten years and then destroyed.

**What will happen to the results?** The results will be seen by the research team. We will also use findings in an anonymized way in publications and presentations to share the information with healthcare professionals, scientists and policymakers and the wider community.

**What are the possible disadvantages of taking part?** I don’t envisage any negative consequences for you in taking part.

**What if there is a problem?** At the end of the procedure, I will discuss with you how you found the experience and how you are feeling. If you subsequently feel distressed, I have included a list of support services below:

**YourMentalHealth**

The YourMentalHealth information line is a phone service you can call any time 24/7.

A member of their team can tell you about:

- - the mental health supports and services available to you
  - how to access different services provided by the HSE and our funded partners
  - opening hours

Freephone: 1800 111 888

The YourMentalHealth information line is not a counselling service.

**Samaritans**

They provide a listening service to anyone who needs it, no matter what you are going through.

Freephone: 116 123

**Pieta House**

They provide support for people who are suicidal and people who self-harm.

Freephone: 1800 247 247

Face-to-face support

For face-to-face support contact your GP.

You can also search this list of mental health supports and services near you.

**Urgent help**

Call 999 or 112 and ask for the ambulance service if you or someone you know needs emergency help.

**Who has reviewed this study?** This study has been reviewed by the Social Research Ethics Committee of UCC.

**Any further queries?** If you need any further information, you can contact me: Grace Phillips ([grace.phillips@ucc.ie](mailto:grace.phillips@ucc.ie)) or contact the HSE contact person, Julieann Lane on (066) 7184535.

If you agree to take part in the study, please sign the consent form overleaf.

**Appendix C**

**Consent Form for Staff and Service Users**

**CONSENT FORM**

**
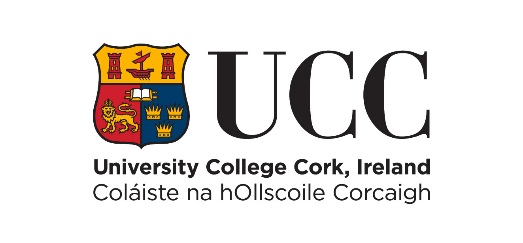
**

I………………………………………agree to participate in the research study, Process Evaluation of the COVID19 Vaccination Programme for the Homeless Population of Cork City.

The purpose and nature of the study has been explained to me in writing.

I am participating voluntarily.

I give permission for my interview with Grace Phillips to be audio-recorded.

I understand that I can withdraw from the study, without repercussions, at any time, whether before it starts or while I am participating.

I understand that I can withdraw permission to use the data within two weeks of the interview, in which case the material will be deleted.

I understand that anonymity will be ensured in the write-up by disguising my identity.

I understand that disguised extracts from my interview may be quoted in the study and any subsequent publications if I give permission below:

(Please tick one box:)

I agree to quotation/publication of extracts from my interview 

I do not agree to quotation/publication of extracts from my interview 

Signed: …………………………………… Date: …………...

PRINT NAME: …………………………………….

**Appendix D**

**Letter of Invitation for Staff and Service Users**

02/11/2021

Dear Sir or Madam

We would like to invite you to take part in an interview as part of a research study conducted by University College Cork. During this interview will ask you questions the about your experience of the COVID19 vaccine rollout to the homeless population of Cork City. The purpose of the study is to investigate the COVID19 vaccine rollout to the homeless population of Cork City, which will help to inform future interventions for vulnerable populations, and achieve a long-term goal of making health services more accessible to difficult to reach groups.

Before you decide if you would like to take part it is important for you to fully understand why the project is being completed and what it will involve. Please take time to carefully read the Information Sheet enclosed.

If you would like to take part, please complete, and return the Informed Consent Declaration form.

Please do not hesitate to contact me if you have any questions.

Yours faithfully,


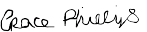


Grace Phillips

School of Public Health, University College Cork

University email: grace.phillips@ucc.ie

**Appendix E**

**Script used for consent for quantitative data to be used:**

“We are doing some work with UCC looking at research into the vaccine uptake by people in homelessness or in drugs and alcohol services.

We would like to look at where people are staying, what ages they are, how many men/women etc. and it will be anonymous, that is, no one will be able to tell who was involved. Your name will not be used.

The plan would be to look at the numbers getting the vaccine from different services and to publish the results.

There is no obligation to be involved”.

**Appendix F**

**Topic Guide for Staff**

**Rollout of the COVID19 Vaccine to the Homeless Population of Cork City: Topic Guide for staff involved in the Pfizer vaccine rollout.**

**Introduction**

Good morning/afternoon. My name is Grace Phillips, and I am from University College Cork.

The objective of this interview is to gain an understanding of your personal experience and opinions on the COVID19 vaccine rollout to the Homeless Population of Cork City. The interviews are part of a research project in which we will collect data on staff and service users of the HSE, Cork Kerry Social inclusion and homeless services . The aim of this study is to investigate the COVID19 vaccine rollout to the homeless population of Cork City, to learn from the experience, to inform future interventions for vulnerable populations, and achieve a long-term goal of making health services more accessible to difficult to reach groups. I would like to thank you again for agreeing to take part in this interview and thereby contributing to the improvement of services for the homeless community.

**INTERVIEWER NOTE: Make it clear to participants that there are no right, or wrong answers and the purpose of the interview is to hear about their opinions and experiences with the COVID19 vaccine rollout.**

Thank you for participating in this interview. I would like to remind you that your participation in this interview is voluntary. This means that you do not have to answer questions that you do not want to, and it means you have the right to terminate and leave the interview at any moment. Everything you say during this interview will be treated as confidential. I would like to ask your permission to record the interview. This is only to allow us to develop a full report of the interview. The report of the interview will not be made available to anyone other than the research team and will only be used for research purposes. This will be anonymous and none of the information you provide for this research will be traceable to you.

**INTERVIEWER NOTE: Check that the interviewee completely understands that all information that they provide is anonymous. Check that the interviewee completely understands that anything discussed, or any information provided is confidential and will not be discussed outside the interview, all information will be used for research purposes only. The interview should not take any more than one hour.**

Do you have any questions?

Do I have permission to proceed with the interview?

**The Covid19 Vaccine Rollout to the Homeless Population**

**Description of the process of the vaccine rollout.**

**First, I would like to discuss the process of rolling out the Pfizer vaccines to the homeless population of Cork City.**

1. Can you tell me about the process?
2. What do you think went well?
3. What do you think did not go well?

**INTERVIEWER NOTE: Discuss all stages of the process from registration to the second dose.**

**Bespoke Service versus Mainstream Services.**

**Secondly, I would like to discuss the mainstream routes to the COVID19 vaccination.**

1. What would have prevented the Homeless Population accessing vaccinations through their GP or through the mainstream service, which was offered to the general population?
2. What changes could be made to mainstream services to prevent these barriers in the future?

**PROBE: Literacy issues with registration etc.**

**Facilitators of the Service.**

**Now I would like to discuss the factors which made this bespoke service so accessible to such a vulnerable group.**

1. What aspects of the service made this accessible to service users?
2. Why do you think you had such a high uptake rate?
3. What were the reasons for using the Pfizer vaccine rather than Jansen?
4. Do you think that there would have been as high an uptake rate if you offered the Jansen vaccine?

**INTERVIEWER NOTE: At the time of vaccination, the Jansen vaccine was not recommended for use in under 50’s in the general population, however it was recommended for use in difficult to reach populations.**

**Implementation into future interventions.**

**Now I would like to discuss how we can transfer the learnings from this bespoke clinic into future health interventions.**

1. Do you think the learnings of this clinic could be incorporated into mainstream services, for example screening?
2. Do you think that the HSE, Cork Kerry Social inclusion and homeless services could use the learning from this clinic to set up future clinics for health interventions?
3. What changes would you make to the process if you were to use it again in the future, for a booster vaccine for example?
4. What aspects of the process would you include in future health interventions?

**INTERVIEWER NOTE: Emphasis on future clinics, interventions which this could be applied to in the past and future.**

**Overall**

1. Can you describe to me your thoughts and feelings about the COVID19 vaccine rollout to the homeless population?
2. Is there anything else you think might be important about the vaccine rollout that we haven’t talked about?

**Closing**

- These were the topics I hoped to discuss with you. Are there any topics you feel are important to the Homeless Community that we did not discuss?
- Do you have any questions for me?
- Do you have any questions about the research?

**NOTE: Remind participants that everything that has been discussed will remain anonymous.**

**Appendix G**

**Topic Guide for Staff**

**Rollout of the COVID19 Vaccine to the Homeless Population of Cork City: Topic Guide for Staff of the HSE, Cork Kerry Social inclusion and homeless services**

**Introduction**

Good morning/afternoon. My name is Grace Phillips, and I am from University College Cork.

The objective of this interview is to gain an understanding of your personal experience and opinions on the COVID19 vaccine rollout to the Homeless Population of Cork City. The interviews are part of a research project in which we will collect data on staff and service users of the HSE, Cork Kerry Social inclusion and homeless services. The aim of this study is to investigate the COVID19 vaccine rollout to the homeless population of Cork City, to learn from the experience, to inform future interventions for vulnerable populations, and achieve a long-term goal of making health services more accessible to difficult to reach groups. I would like to thank you again for agreeing to take part in this interview and thereby contributing to the improvement of services for the homeless community.

**INTERVIEWER NOTE: Make it clear to participants that there are no right, or wrong answers and the purpose of the interview is to hear about their opinions and experiences with the COVID19 vaccine rollout.**

Thank you for participating in this interview. I would like to remind you that your participation in this interview is voluntary. This means that you do not have to answer questions that you do not want to, and it means you have the right to terminate and leave the interview at any moment. Everything you say during this interview will be treated as confidential. I would like to ask your permission to record the interview. This is only to allow us to develop a full report of the interview. The report of the interview will not be made available to anyone other than the research team and will only be used for research purposes. This will be anonymous and none of the information you provide for this research will be traceable to you.

**INTERVIEWER NOTE: Check that the interviewee completely understands that all information that they provide is anonymous. Check that the interviewee completely understands that anything discussed, or any information provided is confidential and will not be discussed outside the interview, all information will be used for research purposes only. The interview should not take any more than one hour.**

Do you have any questions?

Do I have permission to proceed with the interview?

**THE COVID19 VACCINE ROLLOUT TO THE HOMELESS POPULATION**

**Personal Experience**

**I would like to start by talking about your experience with the COVID19 vaccine.**

1. Have you had the COVID19 Vaccine?
2. Can you describe to me your experience with the vaccine?

**PROBE: Include experience from registration right up to the second vaccine.**

**Process Evaluation**

**I would like you to tell me about the positives and negatives of the process used by the HSE, Cork Kerry Social inclusion and homeless services.**

1. Can you describe to me the process of receiving the COVID19 vaccine? What did you have to do?
2. What worked well in this process?
3. What did not work well in this process?

**PROBE: Include positives and negatives of all aspects of the process.**

**Accessibility**

**I would now like to speak about the accessibility of the COVID19 vaccine in general.**

1. What made the vaccine accessible to you?
2. Do you think you would have received the vaccine if the HSE, Cork Kerry Social inclusion and homeless services had not offered you a vaccine?
3. What difficulties might you have had if you had to receive a vaccine from the mainstream service i.e., GP?
4. If you received the second dose of the vaccine, what made it accessible to you?

**PROBE: Touch on registration, organisation, transport, second dose etc.**

**Influence of the use of the Pfizer vaccine on participants decision to receive the vaccine.**

**I would now like to discuss the vaccine.**

1. If you were offered another vaccine (not Pfizer) would you have received the vaccine?
2. What factors influenced you to receive the vaccine? Were your reasons personal or influenced by the process that was used?

**PROBE: Would you have been willing to receive the Jansen, Moderna or AstraZeneca vaccine?**

**How can we apply this to future interventions?**

**I would now like to discuss other health interventions and how this process could be used to make other health interventions more accessible to you. By health interventions I mean services like cancer screening or a booster vaccine.**

1. Are there any changes you would have made to the vaccine rollout to make it more accessible?
2. Can you think of any other health interventions or services that this process could be used for?
3. Have you come into contact with any barriers in the past that prevented you from accessing any other health services? By barriers I mean any problems that have stopped you from using a service.

**PROBE: Are there any services that you have wanted to access in the past but were unable to due to barriers such as transport etc.?**

**Overall**

1. Can you describe to me your thoughts and feelings about the COVID19 vaccine rollout to the homeless population?
2. Is there anything else you think might be important about the vaccine rollout that we haven’t talked about?

**Closing**

- These were the topics I hoped to discuss with you. Are there any topics you feel are important to the Homeless Community that we did not discuss?
- Do you have any questions for me?
- Do you have any questions about the research?

**NOTE:** **Remind participants that everything that has been discussed will remain anonymous.**
